# Actin-associated proteins and cardiomyopathy—the ‘unknown’ beyond troponin and tropomyosin

**DOI:** 10.1007/s12551-018-0428-1

**Published:** 2018-06-05

**Authors:** Elisabeth Ehler

**Affiliations:** 1 Randall Centre for Cell and Molecular Biophysics (School of Basic and Medical Biosciences), London, UK; 2 School of Cardiovascular Medicine and Sciences, British Heart Foundation Research Excellence Centre, King’s College London, Room 3.26A, New Hunt’s House, Guy’s Campus, London, SE1 1UL UK

**Keywords:** Actin-binding proteins, Formin, Cytoskeleton, Cardiomyopathy, Intercalated disc

## Abstract

It has been known for several decades that mutations in genes that encode for proteins involved in the control of actomyosin interactions such as the troponin complex, tropomyosin and MYBP-C and thus regulate contraction can lead to hereditary hypertrophic cardiomyopathy. In recent years, it has become apparent that actin-binding proteins not directly involved in the regulation of contraction also can exhibit changed expression levels, show altered subcellular localisation or bear mutations that might lead to hereditary cardiomyopathies. The aim of this review is to look beyond the troponin/tropomyosin mechanism and to give an overview of the different types of actin-associated proteins and their potential roles in cardiomyocytes. It will then discuss recent findings relevant to their involvement in heart disease.

## Introduction

Two major different types of cardiomyopathy can be defined in human patients, hypertrophic cardiomyopathy (HCM) and dilated cardiomyopathy (DCM; for review, see Seidman and Seidman 2001). While HCM shows obvious signs of myocyte disarray in conventional histology, the phenotype of DCM is more subtle and can usually only be elucidated by immunohistochemistry and electron microscopy (Pluess and Ehler 2015). The major changes in DCM appear to occur at the intercalated disc, the specialised cell-cell contact between cardiomyocytes. These changes lead to an altered molecular composition and include an increased expression of actin-anchoring proteins (Ehler et al. 2001). In addition, signalling molecules such as PKCalpha are recruited to the intercalated disc (Lange et al. 2016). While about 75% of mutations that lead to hereditary HCM are found in the genes encoding for sarcomeric myosin heavy chain (MYH7) and myosin-binding protein-C (MYBPC3; McNally et al. 2013), other components of the myofibrils can be mutated such as the troponins and alpha-tropomyosin (Tardiff 2011). Initially, it was believed that HCM was a disease of the sarcomere. However, with the identification of mutations in more genes that encode for proteins that do not stably associate with myofibrils (Geier et al. 2008), this was probably an over-simplification. Similarly, the hypothesis that hereditary DCM is caused solely by mutations in cytoskeletal proteins had to be abandoned, since mutations in genes that encode for sarcomeric proteins result in this disease phenotype, too (McNally et al. 2013). It may be more the position of the mutation in the molecule or the combination with mutations in other genes that results in a HCM versus a DCM phenotype (McNally and Mestroni 2017; Tardiff 2011).

As far as components of the thin (actin) filaments are concerned, mutations were described for tropomyosin, troponin T, troponin I and troponin C as well as for cardiac actin itself (Hoffmann et al. 2001; Kimura et al. 1997; Olson et al. 1998; Watkins et al. 1995). However, more recently, it was also shown that mutations in actin-interacting proteins that are not directly involved in contraction or its regulation, such as FHOD3, alpha-actinin or filamin C, can cause hereditary cardiomyopathies (Arimura et al. 2013; Girolami et al. 2014; Tucker et al. 2017; Wooten et al. 2013). These reports prompted the writing of this review on actin and its associated proteins beyond the sarcomere.

**Fig. 1:**
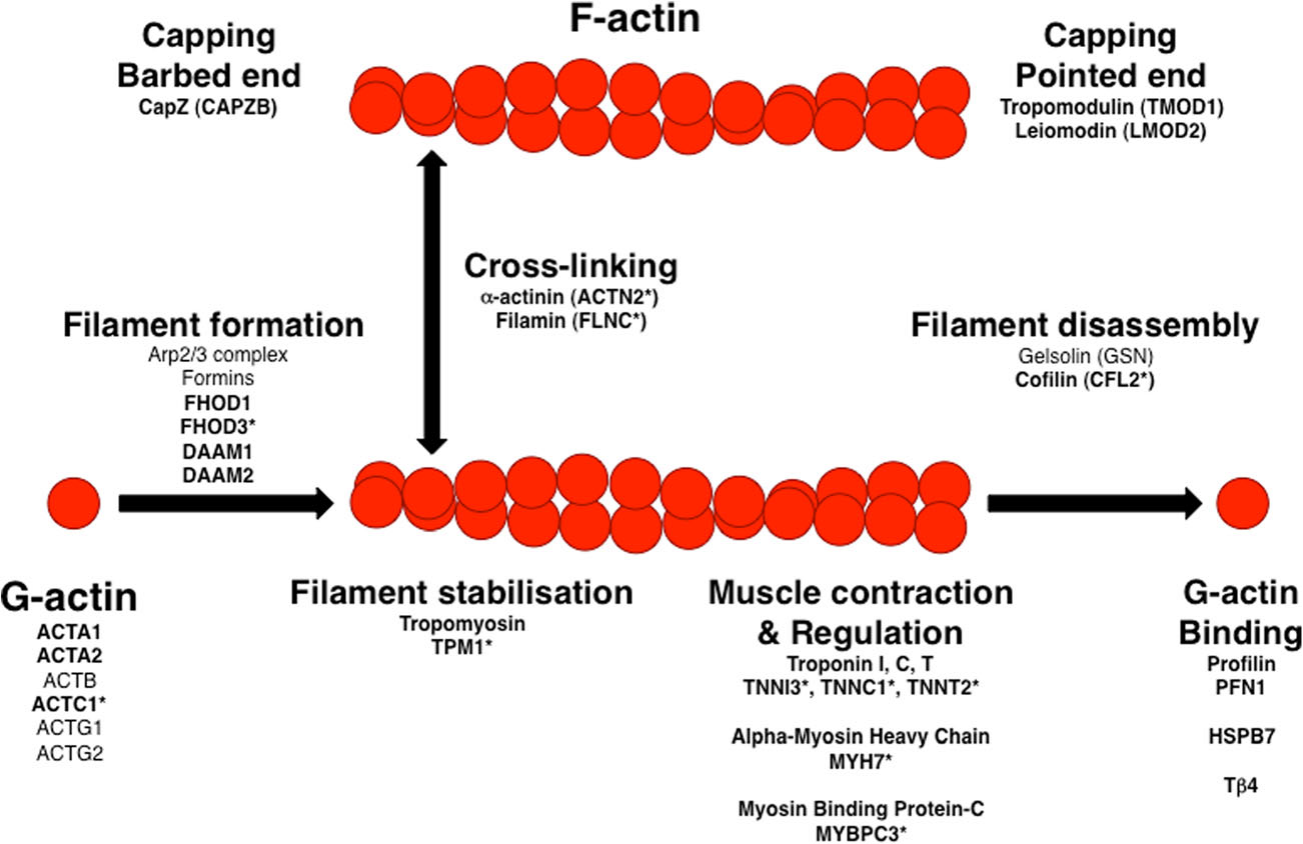
Overview of actin-binding proteins and their effect on actin. Actin-binding proteins can enhance the formation of filaments from G-actin monomers, can stabilise and crosslink these filaments and can also disassemble them. The end of the filaments are termed barbed (plus end) and pointed (minus end) and dissociation of G-actin is prevented by different capping proteins. Disassembly of actin filaments is favoured by members of the gelsolin family. Gene names are given below the roles; names in bold are highly expressed in cardiomyocytes. An asterisk after the name indicates that these genes were shown to bear mutations that can cause hereditary cardiomyopathy

**Fig. 2:**
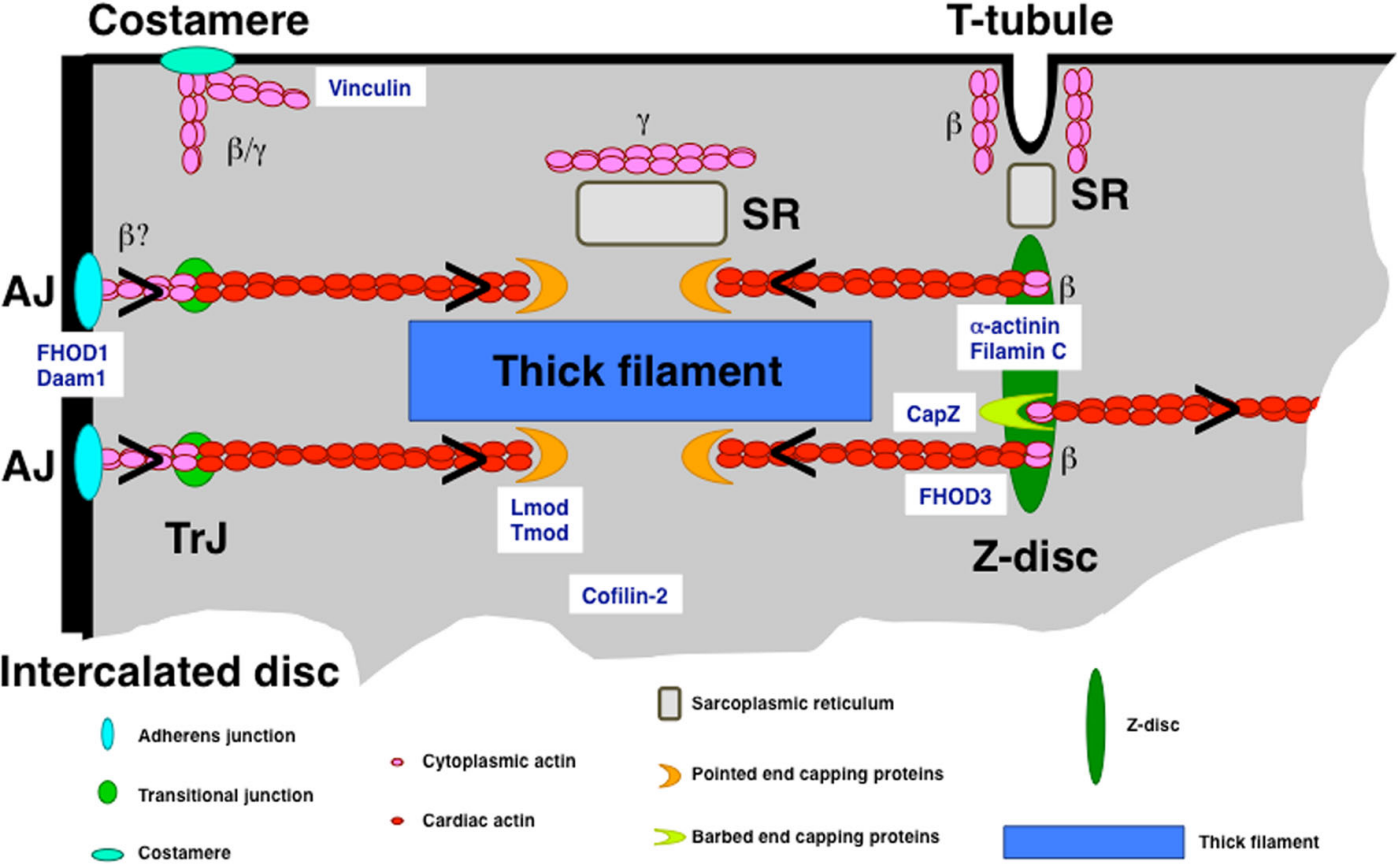
Overview of the different types of actin filaments and subcellular localisation of different actin-associated proteins in a cardiomyocyte. Only one corner of the cell is shown. The legend below shows the different types of complexes, which are mostly represented in a very simplified fashion. Chevrons indicate the orientation of the actin filaments

Actin is a highly conserved eukaryotic protein that exists as six distinct isoforms: alpha-cardiac, alpha-skeletal, alpha-smooth muscle, beta-cytoplasmic, gamma-cytoplasmic and gamma-smooth muscle actin (Vandekerckhove and Weber 1978). Actin monomers (G-actin) can associate to form filaments (F-actin; see Fig. 1) that have the appearance of two helically entwined pearl strings (Hanson and Lowy 1963). However, this is an energetically unfavourable process, which is massively enhanced by factors that promote actin filament formation such as the Arp2/3 complex or members of the formin family (Chesarone and Goode 2009). Once filaments are formed, they can be stabilised laterally via the association of tropomyosin in one of its numerous isoforms (Gunning et al. 2015). Based on their distinct dynamics, the ends of an actin filament are termed plus end (where incorporation of new actin monomers happens; also called barbed end based on the decoration with myosin heads) and minus end (also called pointed end, where actin monomers are lost in the process of treadmilling). These ends can be protected by the association of capping proteins such as CapZ at the barbed end or tropomodulin and leiomodin at the pointed end (Fig. 2). In addition, actin filaments can be crosslinked to meshworks or bundled to parallel filaments and there are severing proteins that lead to their disassembly (for a landmark review on actin-binding proteins, see Pollard and Cooper 1986, and for a more recent review, see dos Remedios et al. 2003).

Adult cardiomyocytes mainly express the alpha-cardiac actin isoform, which is found almost exclusively in the myofibrils, although cytoplasmic actin isoforms are expressed at very low levels and can be detected in the vicinity of membranes and at the Z-disc (Benz et al. 2013; Dwyer et al. 2012; Kee et al. 2009; Tondeleir et al. 2009, Fig. 2). In the early embryonic heart, alpha-cardiac and alpha-smooth muscle actin are co-expressed in the same thin filaments (Ehler et al. 2004). Confocal microscopy suggests that while the length of the cardiac actin filaments is determined quite early, there exists a population of actin filaments that extends beyond the I-band, which may be mainly composed of alpha-smooth muscle actin (Ehler et al. 2004). Currently, it is unknown whether there are mixed actin filament populations and whether the length-determining factor is functional rather than molecular, since tropomodulin and leiomodin are absent at the pointed ends or not expressed at this developmental stage (Ehler et al. 2004; Tsukada et al. 2010). Similar to other foetal marker genes, an upregulation of alpha-smooth and even alpha-skeletal muscle actin can be detected in hypertrophic cardiomyopathy (Copeland et al. 2010; Suurmeijer et al. 2003). An inbred mouse strain, the Balb/c mouse, also has a higher expression level of alpha-skeletal actin and shows increased contractility (Hewett et al. 1994).

## Actin filament assembly and maintenance in cardiomyocytes

As mentioned above, since actin filament assembly is an inefficient process, there is a need for factors that might promote it, especially as the half-life of actin and its associated proteins in a cardiomyocyte range from 3 to 10 days (Martin 1981). The cell migration field has pioneered this research and two main basic ways were identified: (1) filament assembly by the Arp2/3 complex of proteins, which tends to support the formation of filaments at an angle to the mother filaments and (2) filament assembly by members of the formin family, which promote the formation of linear filaments (Chesarone and Goode 2009). Not much is known about Arp2/3 in the heart, but in the skeletal muscle, a role for an Arp2/3 family member, Arpc5L, was shown for the coordination between gamma-actin filaments, the desmin cytoskeleton and nuclear positioning (Roman et al. 2017). More and more of the 15 members of the formin family are characterised as having a role in the heart (Li et al. 2011; Rosado et al. 2014; Taniguchi et al. 2009; reviewed in Randall and Ehler 2014).

Knockout mice for the formin Daam1 mainly reveal a more general role in heart morphogenesis with a noncompaction phenotype and septal defects (Ajima et al. 2015; Li et al. 2011). This is especially interesting, since a recent report showed a potential association of a deletion of a DAAM1 copy with congenital heart disease (Bao et al. 2012). Myofibrils are assembled, but are disorganised, and there may be a problem with their maintenance (Ajima et al. 2015). The major phenotype is seen at the intercalated discs, where cardiomyocyte attachment is severely impaired (Ajima et al. 2015). This is in agreement with localisation data for Daam1 close to the plasma membrane and its potential role in the Wnt effector Dishevelled and thus the planar cell polarity signalling pathway (Li et al. 2011). The phenotype is more severe in Daam1-Daam2 double knockout mice, suggesting a certain redundancy between these proteins.

The formin FHOD3 seems to play a role in early heart development and subsequently in myofibril maintenance. FHOD3 knockout mice did not survive beyond E12.5 and showed hypokinetic ventricles with myofibrillar disarray and Z-disc malformations (Kan-o et al. 2012a). However, a conditional knockout of FHOD3 expression in adult mice did not lead to a lethal phenotype, but just to a mild impairment of cardiac function (Ushijima et al. 2018). Experiments with knockdown of FHOD3 expression in cultured cardiomyocytes also demonstrated a failure to maintain myofibrils and reduced expression levels in samples from human heart failure patients (Iskratsch et al. 2010). Currently, the exact role of FHOD3 in cardiomyocytes is as unclear as its subcellular localisation. We and others detected FHOD3 exclusively at the Z-discs of isolated adult cardiomyocytes and in adult heart tissue from mice and humans (Iskratsch et al. 2010; Rosado et al. 2014), which would fit well with a role as barbed-end facilitator of actin assembly, while others have reported a broader localisation, which overlaps the A-band (Kan-o et al. 2012b). In our hands, this kind of FHOD3 localisation is only detected in the embryonic heart and in cultured neonatal rat cardiomyocytes that are adapting to life in two dimensions in a culture dish (Iskratsch et al. 2010). On the other hand, a recently identified interaction between FHOD3 and MyBP-C, which associates with a subset of the myosin heads, favours the A-band localisation (Matsuyama et al. 2018). Patients with mutations in the FHOD3 gene can develop HCM or DCM (Arimura et al. 2013; Wooten et al. 2013). Potentially, FHOD3 is not firmly integrated into the sarcomere and exerts its role by influencing MyBP-C, which is a regulator of the thick filament on-off state (Kampourakis et al. 2014), or it may affect the ratio of available actin monomers. This could explain its detrimental effect on the activation of the transcription factor SRF in the case of the DCM mutant (Arimura et al. 2013).

## What happens at the anchorage sites of the myofibrils, the intercalated discs?

As mentioned above, the major subcellular changes observed in DCM occur at the intercalated disc. Both in mouse models for this disease and in human DCM samples, we observed increased expression of all proteins involved in anchoring of actin filaments (i.e. the myofibrils in the cardiomyocytes): the transmembrane cadherins, and at the cytoplasmic face the catenins and N-RAP (Ehler et al. 2001; Pluess et al. 2015). The increased width of signal for these proteins that was seen at the intercalated disc by confocal microscopy was due to a higher degree of membrane convolution, as demonstrated by ultrastructural analysis (Wilson et al. 2014). Analysis of the actin signal using the F-actin stain phalloidin in 0.25-μm-thick cryosections also revealed a higher intensity at the intercalated disc in mouse models for DCM, suggesting that the increased amount of actin-anchoring proteins mirrors an increased presence of filamentous actin there (Ehler et al. 2001). Currently, it is not known which protein is involved in generating more filamentous actin at the intercalated disc, but the observation that the formin FHOD1 locates to this subcellular domain (Al Haj et al. 2015) and its signal is also increased in DCM (Dwyer et al. 2014) makes it a promising candidate. FHOD1 was thought to be unable to promote the formation of actin filaments and to act just as an actin capper and actin bundler (Schönichen et al. 2013). However, FHOD1 participates in the nucleation of actin filaments from early integrin clusters in fibroblasts and is associated with integrins in cardiomyocytes (Al Haj et al. 2015; Iskratsch et al. 2013). Recent evidence also shows that FHOD1’s actin polymerising activity depends on the actin isoform and that while it is inactive with sarcomeric actins (which most people use for in vitro polymerisation assays), it does promote filament formation with cytoplasmic actin isoforms (Patel et al. 2018). Filamentous actin leading up from the transitional junction to the intercalated disc does not seem to contain alpha-cardiac actin (Bennett et al. 2006) and may well be composed of cytoplasmic actins (Benz et al. 2013). Thus, FHOD1 could be an important controlling factor. FHOD1 at the intercalated disc is in an active state, since it can be stained with an antibody against a phosphorylated epitope at T1141. However, it remains to be shown whether it indeed plays a role in excessive actin filament synthesis in DCM (Dwyer et al. 2014).

## What happens at the ends of the thin filaments?

Actin-capping proteins determine the length of thin filaments in healthy cardiomyocytes both at the barbed end at the Z-disc and at the pointed end near the inner edges of the H-zone (reviewed by Dwyer et al. 2012; Fowler and Dominguez 2017).

CapZ binds to the barbed ends of thin filaments (Casella et al. 1987) and its dynamics in myocytes is increased by exercise and during hypertrophy (Lin et al. 2013; Lin et al. 2016). Among the signalling pathways that affect CapZ dynamics are PIP2 (phosphatidylinositol-4,5 bisphosphate), phosphorylation by PKC (protein kinase C) and acetylation (Hartman et al. 2009; Lin et al. 2016). CapZ transgenic hearts that express reduced amounts of CapZ are protected against ischemia-reperfusion injury and show alterations in PKC signalling (Yang and Pyle 2012). In addition to its adaptive dynamic behaviour upon cardiomyocyte stress, CapZ also interacts with classical stress signals such as the co-chaperone BAG3 and the small heat shock protein Hsc70 (Hishiya et al. 2010).

Gain of function (overexpression of tropomodulin) and loss of function experiments (interfering with tropomodulin binding) have shown that the tight control of thin filament length at its pointed end is crucial for a healthy cardiomyocyte (Fritz-Six et al. 2003; Gregorio et al. 1995; Sussman et al. 1998) and tropomodulin seems to be the major protein responsible for capping the pointed ends. However, in recent years, a related protein, called leiomodin, was described, which is also needed to maintain myofibrils (Chereau et al. 2008) and results in a DCM phenotype with early postnatal death in knockout mice (Pappas et al. 2015). Interestingly, there seems to be crosstalk between tropomodulin, leimodin2 and an actin-monomer-binding protein, Hspb7, that was reported to be mutated in DCM (Stark et al. 2010). Knockout mice for Hspb7 have longer thin filaments in their sarcomeres that even seem to connect two neighbouring Z-discs and are crosslinked by alpha-actinin. Lmod2 expression is upregulated, suggesting that its uncontrolled activity contributes to the excessive actin filament synthesis and the signal for tropomodulin becomes diffuse (Wu et al. 2017).

## What happens at the Z-discs?

Mutations in several Z-disc proteins are associated with a HCM phenotype (Bos and Ackerman 2010). For example, missense mutations in the gene ACTN2 were described, which encodes the actin-crosslinking protein alpha-actinin, the marker protein for Z-discs (Chiu et al. 2010). Thorough molecular characterisation of these mutations is still under way, but first results indicate that at least in the case of A119T and G111V mutations, the dynamic behaviour of alpha-actinin and its readiness to incorporate into the Z-disc may be affected (Haywood et al. 2016). A second major Z-disc-associated actin-binding protein that was shown to cause cardiomyopathy when mutated is filamin C. Missense mutations of filamin C cause familial restrictive cardiomyopathy and lead to a loss of filamin C signal at the Z-disc (Tucker et al. 2017). Truncating variants of filamin C and its co-chaperone BAG3 are associated with DCM (Janin et al. 2017). Interestingly, BAG3 stimulates filamin transcription and also spatially regulates mTORC1 signalling to simultaneously induce autophagy of damaged filamin and activate protein synthesis upon mechanical stress in cardiomyocytes (Kathage et al. 2017). Again, these results indicate that actin-associated proteins are not just static glue at their respective sites but closely interweave with signalling pathways that are relevant for the cardiomyocyte stress response.

## Proteins involved in actin filament turnover and their role in cardiac disease and repair

Cofilin-2 is a member of the ADF/cofilin family of proteins that acts preferentially at the pointed ends of actin filaments and increases the off-rate by 30-fold (for a review, see dos Remedios et al. 2003). In cultured cardiomyocytes, cofilin-2 was shown to localise towards the M-band region of the sarcomeres, where the pointed ends are found (Kremneva et al. 2014). In a healthy cardiomyocyte in situ, cofilin-2 should not affect the structure of thin filaments too much, since the pointed ends are protected by tropomodulin or leiomodin and cofilin’s depolymerising activity is known to be inhibited by the presence of tropomyosins (dos Remedios et al. 2003). However, when cofilin-2 expression is knocked down in cultured cardiomyocytes, a marked elongation of thin filaments is observed and proper I-band striations are lost (Kremneva et al. 2014). In a mouse model for DCM, the calsarcin knockout mouse, cofilin-2 expression was increased due to a decrease in miRNA miR-301a expression and was subsequently shown to be a direct target for this miRNA (Rangrez et al. 2017). Cofilin-2 function is regulated by phosphorylation, and fasudil, an inhibitor of ROCK (Rho kinase), which has a protective effect against cardiac dysfunction, prevents its phosphorylation and promotes the organisation of actin filaments (Lai et al. 2017). In human idiopathic DCM, aggregates of cofilin-2 in its phosphorylated state were detected in the heart samples of patients (Subramanian et al. 2015). To model reduced cofilin-2 activity, a heterozygous cardiac specific cofilin-2 knockout mouse was generated, which expressed only 40% of cofilin-2 compared to wild-type littermates. These mice displayed dilation and wall thinning of the left ventricle (Subramanian et al. 2015). The reduced contractile function was attributed to disorganised sarcomeres in the heterozygous cofilin-2 mice (Subramanian et al. 2015). These data suggest that cofilin-2 has a regulatory role also in cardiomyocytes and that its expression must be tightly controlled to prevent cardiomyopathy.

Profilin is a protein that sequesters actin monomers and governs their ATP-associated state, leading to a higher affinity for the barbed ends (for a review, see dos Remedios et al. 2003). Interestingly, the barbed ends are classically assumed to be the major site of activity of formins. Formins are characterised by two formin homology (FH) domains, FH1 and FH2. The FH2 domains of two formins dimerise into a doughnut-like structure that forms the business end for formin-promoted actin assembly (Goode and Eck 2007), while the FH1 domain interacts with profilin and may help to shunt profilin-bound actin monomers to the neighbouring FH2 domain. Since profilin dissociates from actin in the presence of PIP and PIP2, the environment of the Z-disc, which is enriched in PIP2 (Pyle et al. 2006; Ribeiro et al. 2014), would be an ideal location to release the actin from profilin and make it available for polymerisation. A recent study has demonstrated that the expression of profilin is increased in a variety of rodent models for hypertrophy in situ and in vitro, but decreased in end-stage heart failure patients (Kooij et al. 2016). In vivo experiments in Drosophila showed that overexpression of profilin leads to longer thin filaments than in control strains and results in a functional phenotype resembling dilated cardiomyopathy (Kooij et al. 2016). Knockdown of profilin expression in cultured cardiomyocytes prevented their hypertrophic response, probably by impaired activation of ERK1/ERK2 signalling (Kooij et al. 2016). In conclusion, profilin appears likely to be crucial for hypertrophic growth in the heart, potentially by delivering actin monomers for assembly by members of the formin family.

Another small actin monomer-binding protein, thymosin beta 4 (Tbeta4), has recently entered the limelight by its ability to enhance cardiac repair in the adult heart following injury (Smart et al. 2011). Tbeta4 was administered to the mice by intraperitoneal injection and somehow seemed to activate a population of stem cells in the epicardial surface of the heart that differentiated into blood vessels but also to a much lower extent into cardiomyocytes. The exact role of Tbeta4 in cardiomyocytes is somewhat unclear at the moment, since Tbeta4 knockout mice had no cardiac phenotype (Banerjee et al. 2012). Therefore, its contribution to improved cardiac repair may be mainly due to its enhancement of vascularisation of the injured heart. On the other hand, another group reported shorter sarcomere length, expression of shorter titin isoforms and a limited contractile reserve in their Tbeta4 knockout mice, suggesting that Tbeta4 may be somehow involved in the regulation of alternative splicing of titin, potentially via RBM20 (Guo et al. 2012; Smart et al. 2017).

## Concluding remarks

With the advance of next-generation sequencing, it can be expected that many more point mutations will be identified in actin-associated proteins in patients with cardiomyopathy, whose contribution to the observed functional phenotypes will have to be validated. However, it is obvious that the actin cytoskeleton is dynamic and that subtle changes that affect this dynamics and its amount will alter the function of a cardiomyocyte.
